# Two new species of *Favolaschia* (*Mycenaceae*, *Agaricales*) from southern and eastern China

**DOI:** 10.3897/mycokeys.143.212718

**Published:** 2026-11-03

**Authors:** Yu-Han Wang, Zi-Wei Zheng, Qian-Xin Guan, Wan-Ying Li, Shu-Bin Liu, Guang-Yu Zeng, Fang Wu

**Affiliations:** 1 School of Ecology and Nature Conservation, Beijing Forestry University, Beijing 100083, China School of Ecology and Nature Conservation, Beijing Forestry University Beijing China; 2 Guangxi Forestry Science Research Institute, Nanning 530002, China Guangxi Forestry Science Research Institute Nanning China

**Keywords:** *
Mycenaceae
*, phylogeny, taxonomy, wood-decaying fungi

## Abstract

Two new species in *Favolaschia*, namely *F. guangxiensis* and *F. microcarpa*, are illustrated and described from southern and eastern China, based on morphological characteristics and phylogenetic analyses of a combined ITS+nLSU dataset. *F. guangxiensis* is characterized by its greyish-white basidiomata when fresh, broadly ellipsoid basidiospores measuring 6.2–11 × 4.1–9 μm and inhabiting dead bamboo; *F. microcarpa* is characterised by its very small (1–3 × 1–2 mm) and white basidiomata, broadly ellipsoid to subglobose basidiospores measuring 6.3–11 × 5.1–9 μm and inhabiting *Miscanthus* sp. and *Trachycarpus fortunei*. The differences between the two new species and their morphologically similar and phylogenetically related species are discussed.

## Introduction

*Favolaschia* (Pat.) Pat., with *F. gaillardii* (Pat.) Pat. as the type species (Patouillard 1887), belongs to *Mycenaceae*, *Agaricales* (Moncalvo et al. 2002; Bodensteiner et al. 2004). The genus is characterised by gelatinous basidiomata, poroid hymenophores, hyphal structure usually with clamp connections, variable cystidia in shape, and amyloid basidiospores (Singer 1945, 1974; Clémençon 1977; Gillen et al. 2012; Capelari et al. 2013; Magnago et al. 2013; Zhang and Dai 2021; Zhang et al. 2023; Lu et al. 2025). *Favolaschia* mostly grows on decaying tissues of monocotyledons and ferns, and causes white rot (Singer 1974; Gillen et al. 2012; Zhang et al. 2023). Species of *Favolaschia* exhibit a global distribution (excluding Antarctica) and prefer humid climates, with the highest species diversity in subtropical and tropical regions (Singer 1974; Gillen et al. 2012; Magnago et al. 2013; Zhang et al. 2024). As of 27 July 2026, a total of 162 taxonomic records of *Favolaschia* are listed in Index Fungorum (http://www.indexfungorum.org), and 183 records in MycoBank (https://www.mycobank.org/), but only around 64 species are accepted (Johnston et al. 2006; Gillen et al. 2012; Magnago et al. 2013; Zhang et al. 2024; Lu et al. 2025).

Singer (1974) and Johnston et al. (2006) indicated that *Favolaschia* has a complicated taxonomic history. The generic name *Favolaschia* was first published by Patouillard (1887) as a section of *Laschia* Fr., later elevated to generic rank (Patouillard and Lagerheim 1892). Based on morphological studies, Singer (1974) divided the genus into two sections: section *Favolaschia* Singer and section *Anechinus* Singer. Taxa of *F.* sect. *Favolaschia* are mainly distinguished by orange, yellow, red, lateritious or pink pilei, and the presence of acanthocytes in the pileipellis. In contrast, taxa of F. sect. Anechinus lack acanthocystidia, with these structures replaced by diverticulate hyphae. The two sections were supported by earlier phylogenetic analyses (Johnston et al. 2006; Gillen et al. 2012; Magnago et al. 2013), but they didn’t form two monophyletic clades in the later phylogenies (Zhang et al. 2023, 2024; Lu et al. 2025).

In recent years, the genus *Favolaschia* has gradually attracted researchers’ attention and systematic study of *Favolaschia* has been updated in China. Zhang and Dai (2021) performed phylogenetic analyses of *Favolaschia* based on extensive sampling, and revealed four new species within the *F. calocera* complex. Zhang et al. (2023) re-summarized the species diversity, phylogenetic relationships, divergence time and geographical distribution of *Favolaschia* using abundant samples from wide regions of China, and confirmed that 16 species of *Favolaschia* have been distributed in China. Based on morphological and phylogenetic analyses of extensive Chinese specimens, Lu et al. (2025) described one new species in Favolaschia sect. Anechinus from China. Currently, 20 previously published species have been recorded in China, namely, *F. brevibasidiata* Y. Zhang & C. Dai, *F. brevistipitata* Y. Zhang & C. Dai, *F. longistipitata* Y. Zhang & C. Dai, *F. minutissima* Y. Zhang & C. Dai, *F. bannaensis* Q.Y. Zhang & Y.C. Dai, *F. crassipora* Q.Y. Zhang & Y.C. Dai, *F. flabelliformis* Q.Y. Zhang & Y.C. Dai, *F. imbricata* Q.Y. Zhang et al., *F. manipularis* (Berk.) Teng, *F. miscanthi* Q.Y. Zhang et al., *F. peziziformis* (Berk. and M.A. Curtis) Kuntze, *F. pustulosa* (Jungh.) Kuntze, *F. rigida* Q.Y. Zhang & Y.C. Dai, *F. semicircularis* Q.Y. Zhang & Y.C. Dai, *F. sinarundinariae* Q.Y. Zhang et al., *F. subpustulosa* Q.Y. Zhang & Y.C. Dai, *F. tenuissima* Q.Y. Zhang & Y.C. Dai, *F. tephroleuca* Q.Y. Zhang & Y.C. Dai, *F. tonkinensis* (Pat.) Kuntze and *F. variistipitata* W.H. Lu, Tibpromma & Karun; the first four species belong to the *F. calocera* complex of the *F.* sect. *Favolaschia*; while the others belong to the F. sect. Anechinus (Zhang and Dai 2021; Zhang et al. 2023, 2024; Lu et al. 2025).

Wood-inhabiting fungi play important roles in decomposing lignin, cellulose and hemicellulose, thereby driving material cycling and carbon storage in forest ecosystems (Dong et al. 2024, 2025; Wang et al. 2024; Song et al. 2025; Yang et al. 2025; Zhao et al. 2025). During investigations of wood-inhabiting fungi in southern China, six specimens morphologically corresponding with *Favolaschia* were collected, and they were identified as two new species in Favolaschia sect. Anechinus based on morphological characters and phylogenetic analyses using a combined dataset of ITS1-5.8S-ITS2-nLSU sequences. Accordingly, the two species are described and illustrated in this study, which expands our knowledge of species diversity of Favolaschia sect. Anechinus in China.

## Materials and methods

### Morphological studies

The studied specimens were collected from Guangxi Autonomous Region and Zhejiang Province in southern and eastern China. They were deposited at the Herbarium of Beijing Forestry University (BJFC). Macromorphological descriptions are based on field notes and laboratory observations. Micromorphological observations and descriptions were carried out on sections mounted in 5% potassium hydroxide solution (KOH) and 2% phloxine B (C_20_H_2_Br_4_Cl_4_Na_2_O_5_), using a Nikon Eclipse 80i microscope with phase-contrast illumination at magnifications up to 1000×. Colour terminology follows Kornerup and Wanscher (1978) and Petersen (1996). Microstructures were photographed using a Nikon Digital Sight DS-L3 camera. Additional reagents, including cotton blue and Melzer’s reagent, were used for micromorphological observations, following the methods of Zhang et al. (2023). The following abbreviations are used in the descriptions: KOH = 5% potassium hydroxide solution; IKI = Melzer’s reagent; IKI+ = amyloid reaction positive; CB = cotton blue; CB− = non cyanophilous; L = arithmetic mean of spore length; W = arithmetic mean of spore width; Q = length/width ratio (L/W); n = total number of spores measured from the indicated number of specimens.

### DNA extraction, PCR amplification and sequencing

A cetyl trimethylammonium bromide (CTAB) rapid plant genome extraction kit (Aidlab Biotechnologies, Co., Ltd., Beijing, China) was used to obtain DNA products from voucher specimens following the manufacturer’s instructions with some modifications (Wu et al. 2020, 2022; Dong et al. 2024; Yang et al. 2025). The following primer pairs were used to amplify the DNA: ITS5 and ITS4 for the internal transcribed spacer (ITS) region (White et al. 1990) and LR0R and LR7 for the nuclear large subunit (nLSU) rDNA gene (Vilgalys and Hester 1990).

The PCR procedure for ITS was as follows: pre-denaturation at 95 °C for 4 min; 34 cycles of denaturation at 94 °C for 40 s, annealing at 54 °C for 45 s, and extension at 72 °C for 1 min; final extension at 72 °C for 10 min. The PCR procedure for nLSU was as follows: pre-denaturation at 94 °C for 1 min; 34 cycles of denaturation at 94 °C for 30 s, annealing at 50 °C for 1 min, and extension at 72 °C for 1.5 min; final extension at 72 °C for 10 min (Dong et al. 2024; Wang et al. 2024; Song et al. 2025). The PCR products were purified and sequenced by the Beijing Genomics Institute (BGI), Beijing, China. All newly generated sequences in this study were deposited in the GenBank database (http://www.ncbi.nlm.nih.gov/genbank/; Sayers et al. 2024), with accession numbers detailed in Table 1.

**Table 1.** Names, voucher numbers, countries and corresponding GenBank numbers of the taxa used in the phylogenetic analyses of *Favolaschia*.

**Species**	**Specimen no**.	**Locality**	**GenBank accession number**	
			**ITS no**.	**nLSU no**.
*Favolaschia andina*	KG0025	Panama	HM246678	HM246679
*F. aurantiaca*	FK2047	Brazil	JX987670	–
*F. aurantiaca*	KG0013	Panama	–	HM246676
*F. auriscalpium*	Isolate 5	–	KY649461	–
*F. auriscalpium*	TH1018	Guyana	DQ026241	–
*F. austrocyatheae*	PDD75609	New Zealand	NR132809	–
*F. austrocyatheae*	PDD75609	New Zealand	DQ026257	–
*F. bannaensis*	Dai 22587	Yunnan, China	ON870497	ON870473
*F. bannaensis*	Dai 22589	Yunnan, China	ON870498	ON870474
*F. bannaensis*	Dai 22590	Yunnan, China	ON870499	ON870475
*F. brevibasidiata*	Cui 6573	Hainan, China	MZ661794	–
*F. brevibasidiata*	JM98186	Yunnan, China	DQ026239	–
*F. brevistipitata*	Dai 19780	Yunnan, China	MZ661772	MZ661742
*F. brevistipitata*	Dai 19855	Yunnan, China	MZ661773	MZ661743
*F. brevistipitata*	Dai 19856	Yunnan, China	MZ661774	MZ661744
*F. calocera*	PC99060	Madagascar	DQ026252	–
*F. calocera*	PC99497	Madagascar	DQ026253	–
*F. cinnabarina*	Isolate 4421	Brazil	JX987669	–
*F. cinnabarina*	RVPR82	–	–	AF261416
*F. claudopus*	Dai 18656	Australia	MZ661775	MZ661735
*F. claudopus*	Dai 18663	Australia	MZ661776	MZ661734
*F. claudopus*	SR346	Kenya	DQ026237	–
*F. claudopus*	PDD74554	New Zealand	DQ026251	–
*F. claudopus*	PDD75323	New Zealand	DQ026248	–
*F. claudopus*	PDD75686	New Zealand	DQ026249	–
*F. claudopus*	DUKE2952	New Zealand	DQ026238	–
*F. crassipora*	Dai 19769	Yunnan, China	ON870500	ON870476
*F. crassipora*	Dai 19871	Yunnan, China	ON870501	ON870477
*F. cyatheae*	PDD75316	New Zealand	NR132808	–
*F. dealbata*	KG0015	Panama	–	HM246677
*F. flabelliformis*	Dai 20010	Yunnan, China	ON870502	ON870478
*F. flabelliformis*	Dai 20016	Yunnan, China	ON870503	–
*F. flabelliformis*	Dai 24354	Yunnan, China	OR271272	OR260441
***F. guangxiensis***	**Wu 3637**	**Guangxi, China**	**PZ850371**	**PZ850377**
***F. guangxiensis***	**Wu 3705**	**Guangxi, China**	**PZ850372**	**PZ850378**
***F. guangxiensis***	**Wu 3704**	**Guangxi, China**	**PZ850373**	**PZ850379**
*F. heliconiae*	KG0026	Panama	–	HM246680
*F. imbricata*	Dai 24702	Guangdong, China	OR334876	–
*F. imbricata*	Dai 24703	Guangdong, China	OR334877	OR334881
*F. imbricata*	Dai 24704	Guangdong, China	OR334878	–
*F. longistipitata*	Dai 13221	Yunnan, China	MZ661777	–
*F. longistipitata*	Dai 13226	Yunnan, China	MZ661778	–
*F. longistipitata*	Cui 11128	Yunnan, China	MZ661779	–
*F. longistipitata*	Dai 17597	Yunnan, China	MZ661780	–
*F. longistipitata*	Dai 17598	Yunnan, China	MZ661781	–
*F. longistipitata*	Dai 17601	Yunnan, China	MZ661782	–
*F. longistipitata*	Dai 19799	Yunnan, China	MZ661784	MZ661739
*F. longistipitata*	Dai 19893	Yunnan, China	MZ661785	MZ661740
*F. longistipitata*	Dai 20019	Yunnan, China	MZ661786	MZ661741
*F. longistipitata*	Dai 20328	Yunnan, China	MZ661787	–
*F. longistipitata*	Dai 20341	Yunnan, China	MZ661788	–
*F. longistipitata*	Dai 20355	Yunnan, China	MZ661789	–
*F. luminosa*	Gallegos 5324	Bolivia	PP488378	PP488382
*F. luminosa*	Gallegos 5457	Bolivia	PP488379	PP488383
*F. luteoaurantiaca*	Isolate 4475	–	JX987667	–
*F. luteoaurantiaca*	SP445750	Brazil	NR132874	–
*F. macropora*	KG0027	Panama	NR132845	HM246682
*F. manipularis*	Dai 20612	Yunnan, China	MZ801776	MZ914395
*F. manipularis*	Dai 20653	Yunnan, China	MZ801777	–
***F. microcarpa***	**Wu 3254**	**Zhejiang, China**	**PZ850374**	**PZ850380**
***F. microcarpa***	**Wu 3255**	**Zhejiang, China**	**PZ850375**	**PZ850381**
***F. microcarpa***	**Wu 4193**	**Zhejiang, China**	**PZ850376**	**PZ850382**
*F. minutissima*	JM98372	Thailand	DQ026240	–
*F. minutissima*	Dai 10753	Hainan, China	MZ661790	–
*F. minutissima*	Dai 20085	Hainan, China	MZ661791	MZ661736
*F. minutissima*	Dai 20086	Hainan, China	MZ661792	MZ661738
*F. minutissima*	Dai 20088	Hainan, China	MZ661793	MZ661737
*F. miscanthi*	Dai 24652	Guangxi, China	OR334879	OR334883
*F. miscanthi*	Dai 24653	Guangxi, China	OR334880	OR334884
*F. peziziformis*	ICMP15757	Japan	DQ026255	–
*F. peziziformis*	PDD67440	New Zealand	–	AY572008
*F. pustulosa*	PDD75686	New Zealand	DQ026254	–
*F. pustulosa*	Dai 19758	Yunnan, China	MT292325	MT293226
*F. rigida*	Dai 18566A	Guangxi, China	ON870504	ON870479
*F. rigida*	Dai 20764	Yunnan, China	ON870505	ON870480
*F. rigida*	Dai 22614	Fujian, China	ON870506	–
*F. semicircularis*	Dai 19725	Guangdong, China	ON870507	ON870481
*F. semicircularis*	Dai 19923	Yunnan, China	ON870508	ON870482
*F. semicircularis*	Dai 19936	Yunnan, China	ON870509	ON870483
*F. semicircularis*	Dai 19939	Yunnan, China	ON870510	–
*F. semicircularis*	Dai 19980	Yunnan, China	ON870511	ON870484
*F. semicircularis*	Dai 19981	Yunnan, China	ON870512	ON870485
*F. semicircularis*	Dai 22290	Zhejiang, China	ON870513	ON870486
*F. semicircularis*	Dai 22298	Zhejiang, China	ON870515	ON870488
*F. semicircularis*	Dai 22302	Zhejiang, China	ON870516	–
*F. semicircularis*	Dai 23702	Hunan, China	OR271273	OR260442
*F. semicircularis*	Dai 24689	Guangxi, China	OR271274	OR260443
*F. semicircularis*	Dai 24748	Jiangxi, China	OR271275	OR260444
*F. semicircularis*	Dai 24687	Guangxi, China	OR271276	OR260445
*F. semicircularis*	Dai 22383	Fujian, China	ON870517	–
*F. sinarundinariae*	Dai 26115	Sichuan, China	OR575908	OR575906
*F. sinarundinariae*	Dai 26116	Sichuan, China	OR575909	OR575907
*F. sinarundinariae*	Dai 26123	Sichuan, China	OR855969	OR855975
*F. sinarundinariae*	Dai 26129	Sichuan, China	OR855970	–
*F. sinarundinariae*	Dai 26130	Sichuan, China	OR855971	–
*F. sinarundinariae*	Dai 26131	Sichuan, China	OR855972	–
*F. sinarundinariae*	Dai 26134	Sichuan, China	OR855973	OR855976
*F. sinarundinariae*	Dai 26140	Sichuan, China	OR855974	OR855977
*Favolaschia* sp. 1	DUKE2708	Australia	DQ026234	–
*Favolaschia* sp. 1	DUKE2876	Australia	DQ026235	–
*Favolaschia* sp. 1	DUKE3195	Papua New Guinea	DQ026236	–
*Favolaschia* sp. 2	Isolate 4550	Panama	JX987668	–
*F. sprucei*	TH6418	Guyana	DQ026246	–
*F. subpustulosa*	Dai 20719	Yunnan, China	ON870518	ON870489
*F. tenuissima*	Dai 22072	Hainan, China	ON870520	ON870491
*F. tenuissima*	Dai 22071	Hainan, China	ON870519	ON870490
*F. tephroleuca*	Dai 22282	Yunnan, China	ON870521	ON870492
*F. tephroleuca*	Dai 22288	Chongqing, China	ON870522	ON870493
*F. tonkinensis*	Dai 21955	Hainan, China	ON870523	ON870494
*F. tonkinensis*	Dai 21956	Hainan, China	ON870524	ON870495
*F. tonkinensis*	Dai 21964	Hainan, China	ON870525	–
*F. tonkinensis*	Dai 21965	Hainan, China	ON870526	ON870496
*F. tonkinensis*	Dai 21966	Hainan, China	ON870527	–
*F. tonkinensis*	Dai 19704	Guangdong, China	OR271277	OR260446
*F. tonkinensis*	BCC 18686	–	MN093316	MN093317
*F. tonkinensis*	JM98229	Yunnan, China	DQ026247	–
*F. varariotecta*	DUKE3893	Puerto Rico	DQ026243	–
*F. varariotecta*	DUKE4038	Puerto Rico	DQ026244	–
*F. variistipitata*	GMB1061	Yunnan, China	PQ481955	PQ481957
*F. variistipitata*	GMB1062	Yunnan, China	PQ481956	PQ481958
*F. xtbgensis*	HKAS 121667	Yunnan, China	OL413048	OL413044
*F. xtbgensis*	HKAS 121975	Yunnan, China	OL413036	OL413035
*Mycena seminau*	ACL136	Malaysia	KF537250	KJ206952
*Mycena seminau*	ACL308	Malaysia	KF537252	KJ206964

Notes: Newly-generated sequences are in bold; “–” represents missing data.

### Phylogenetic analyses

The newly-generated sequences were checked for ambiguous bases and assembled using BioEdit. The new and reference sequences retrieved from GenBank through BLAST searches (Table 1) were partitioned into ITS1, 5.8S, ITS2 and nLSU. *Mycena seminau* A.L.C. Chew & Desjardin was selected as the outgroup (Zhang et al. 2023, 2024). All sequences were aligned using the G-INS-i iterative alignment algorithm of MAFFT v.7.526 (Katoh et al. 2017; http://mafft.cbrc.jp/alignment/server/), followed by manual adjustment and refinement in BioEdit v.7.0.5.3 and Mesquite 3.70 (Hall 1999). Partitioned alignments were concatenated using Mesquite v.3.70. The phylogenetic tree was constructed using the concatenated ITS1+5.8S+ITS2+nLSU dataset and phylogenetic analysis was conducted through Maximum Likelihood (ML) and Bayesian Inference (BI). All sequences analysed in this study are listed in Suppl. material 1.

Maximum likelihood (ML) analysis was performed using the RaxML-HPC BlackBox tool on the CIPRES Science Gateway platform, with RAxML v.7.2.8 under the GTR+I+G model of site substitution, including estimation of gamma-distributed rate heterogeneity and a proportion of invariant sites (Stamatakis 2006). Statistical support values were obtained by using rapid bootstrapping with 1000 replicates, with default settings for other parameters.

Bayesian inference (BI) analysis was conducted with MrBayes v.3.2.7 (Ronquist and Huelsenbeck 2003). The best-fit partitioning scheme and substitution model were determined by using ModelFinder v.2.2.0 (Kalyaanamoorthy et al. 2017) using the greedy algorithm, with linked branch lengths and the AICc criterion. Four Markov chain Monte Carlo chains (one cold) were constructed for 15,000,000 generations, with sampling every 1000 generations. Convergence was assessed as the standard deviation of split frequencies < 0.01. The first quarter of the trees, which represented the burn-in phase of the analyses, were discarded and the remaining trees were used to calculate posterior probabilities (BPP) in the majority rule consensus tree.

Phylogenetic trees were visualized using FigTree 1.4.4 (Rambaut 2018). A branch was considered strongly supported if it met both of the following criteria: maximum likelihood bootstrap support (BS) ≥ 75% and Bayesian posterior probability (BPP) ≥ 0.95.

## Results

### Phylogenetic analyses

**Figure 1.**
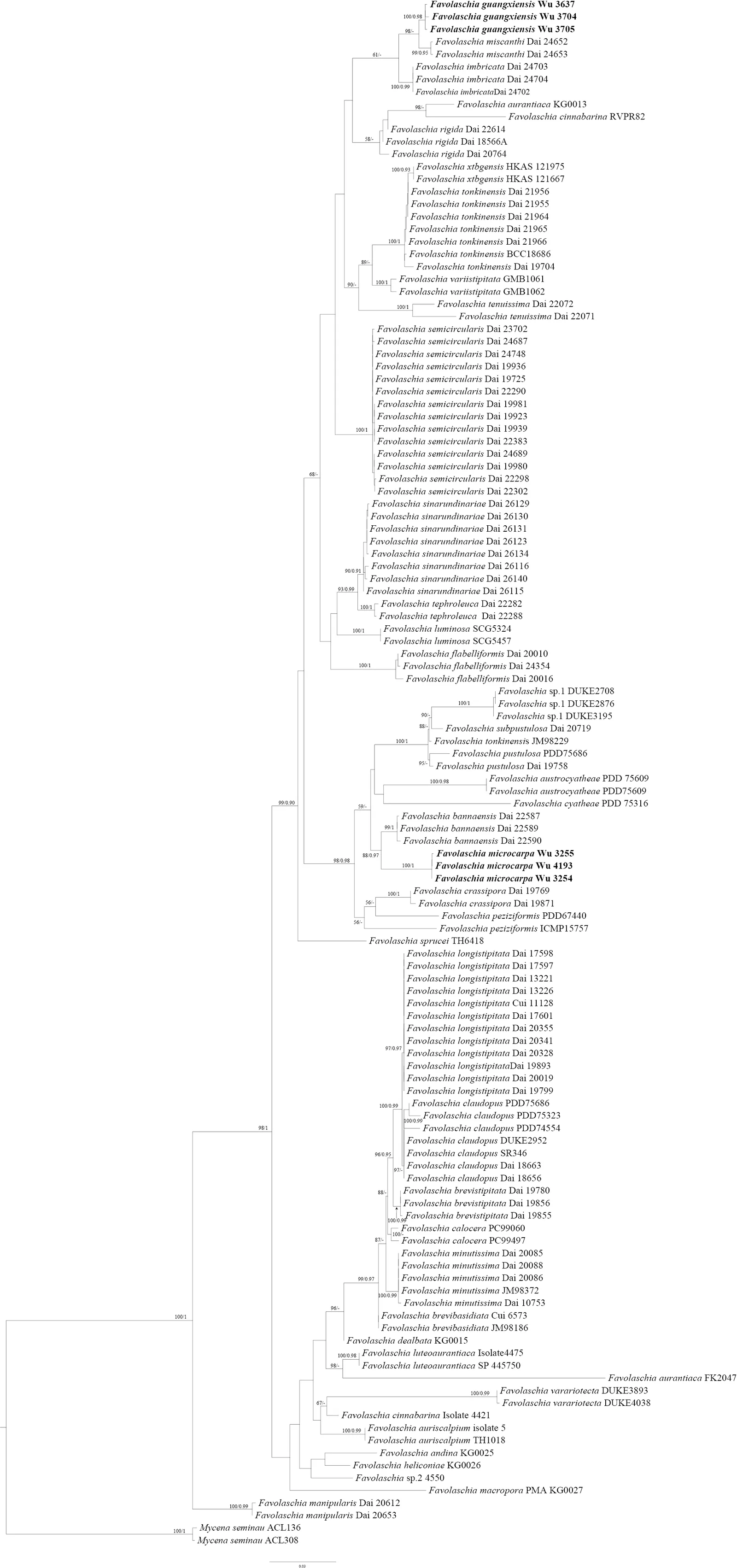
Maximum Likelihood (ML) tree illustrating the phylogeny of *Favolaschia*, based on a combined ITS1-5.8S-ITS2-nLSU dataset. Branches are labelled with maximum-likelihood bootstrap values (ML) equal to or higher than 50% and Bayesian Posterior Probabilities (BPPs) equal to or higher than 0.90. *Mycena seminau* was selected as the outgroup.

In the phylogenetic analysis of *Favolaschia*, the combined ITS1-5.8S-ITS2-nLSU dataset included sequences from 124 fungal collections representing 40 taxa. The dataset had an aligned length of 1679 characters, of which 1,229 (73.20%) were constant, 107 (6.37%) were variable and parsimony-unin formative, and 343 (20.43%) were parsimony-informative. *Mycena seminau* was used as the outgroup (Fig. 1). The substitution models selected by ModelFinder for Bayesian inference were as follows: ITS1: GTR+F+G4; ITS2: GTR+F+I+G4; 5.8S: GTR+F; nLSU: K2P+I+G4. Bayesian inference was performed using MrBayes v3.2.7 with partition-specific models implemented by setting nst = 6 for ITS1, ITS2, and 5.8S, and nst = 2 for nLSU. Among-site rate variation was modeled using rates = gamma for ITS1, rates = invgamma for ITS2 and nLSU, and rates = equal for 5.8S, with four discrete gamma categories where applicable. Nucleotide frequencies were fixed to empirical estimates for ITS1, ITS2, and 5.8S, and to equal frequencies for nLSU. Model parameters were unlinked across partitions, and relative substitution rates were allowed to vary among partitions. The average standard deviation of split frequencies in the Bayesian inference (BI) analysis was 0.008986, indicating good topological convergence.

Topologies obtained from maximum likelihood (ML) and Bayesian inference (BI) were largely congruent; therefore, only the ML tree is presented, with branch support values labeled as ML bootstrap (≥50%) and Bayesian posterior probability (BPP ≥ 0.90). Phylogenetic trees reconstructed using Bayesian and Maximum likelihood methods revealed that our newly collected specimens formed two new highly supported lineages (support values 100/0.98 and 100/1, respectively). Our three specimens (Wu 3705, Wu 3704 and Wu 3637), formed an independent, strongly supported lineage (100/0.98), namely *F. guangxiensis*, which is closely related to *F. miscanthi* Q.Y. Zhang, L.S. Bian, F. Wu & Y.C. Dai. Other three specimens (Wu 3254, Wu 3255 and Wu 4193), formed another independent, strongly supported lineage (100/1), namely *F. microcarpa*, which is closely related to *F. bannaensis* Q.Y. Zhang & Y.C. Dai.

## Taxonomy

### Favolaschia guangxiensis

Taxon classificationFungiAgaricalesMycenaceae

Yu H. Wang, Z.W. Zheng & F. Wu

4BCBE1CA-6636-522F-AEA7-1D4508437DBB

865072

Figs 2, 3, 4

#### Diagnosis.

It is characterized by its greyish-white basidiomata when fresh, broadly ellipsoid basidiospores measuring 6.2–11 × 4.1–9 μm and inhabiting dead bamboo.

**Figure 2.**
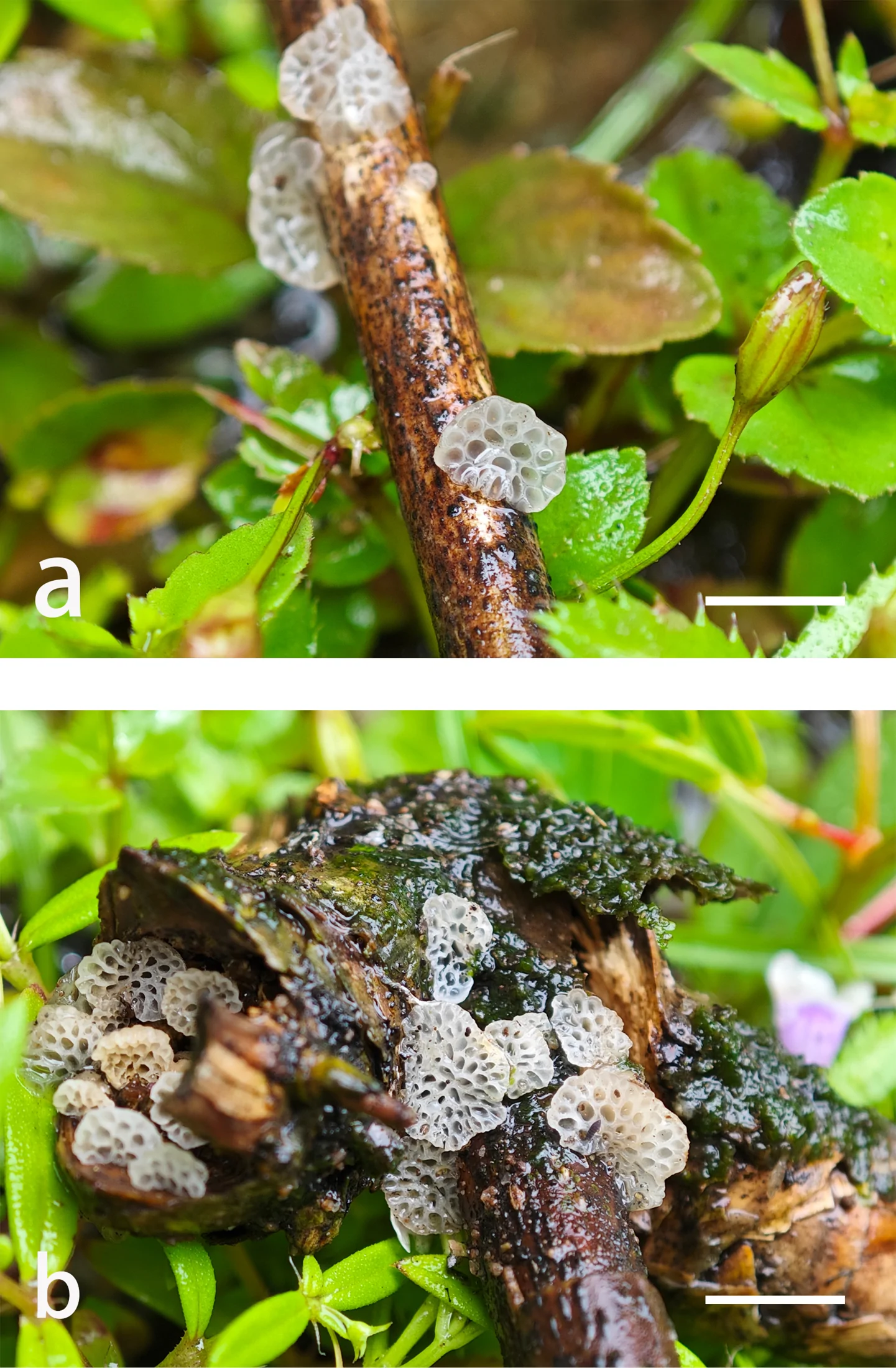
Basidiomata of *Favolaschia guangxiensis*. **a**. Wu 3704 (holotype); **b**. Wu 3705. Scale bars: 1 cm (**a, b**).

**Figure 3.**
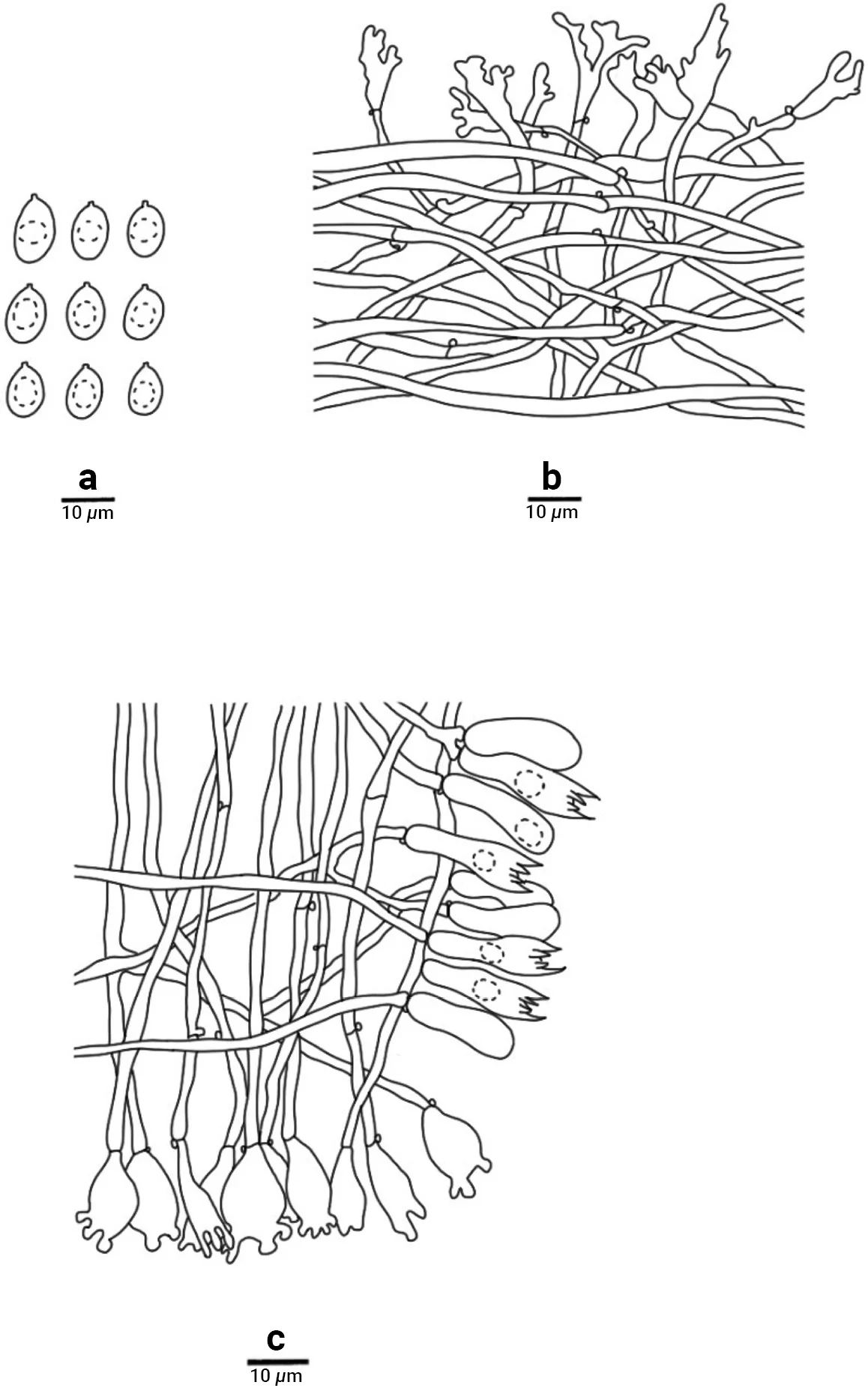
Microscopic structures of *Favolaschia guangxiensis* (holotype). **a**. Basidiospores; **b**. Hyphae and terminal cells from pileipellis; **c**. A section of tube trama, including basidia, basidioles and cheilocystidia.

**Figure 4.**
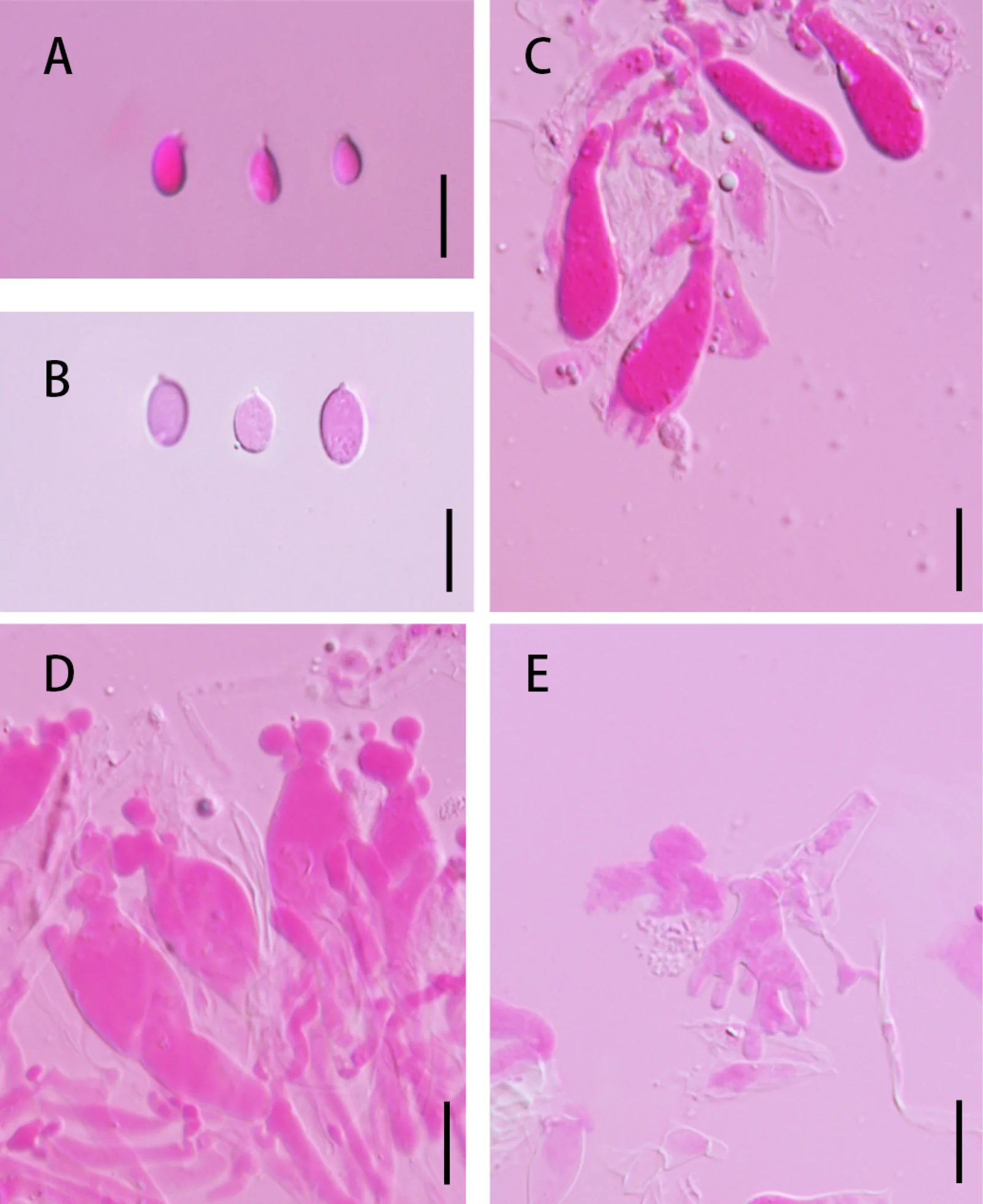
Microscopic structures of *Favolaschia guangxiensis* (holotype). **A, B**. Basidiospores; **C**. A section of tube trama, including basidia and basidioles; **D**. Cheilocystidia; **E**. Terminal cells of pileipellis.

#### Holotype.

China • Guangxi Autonomous Region, Qinzhou, Bazhaigou Scenic Area, 22.1049°N, 108.2543°E, 74 m a.s.l.; 23 July 2025, on dead bamboo, F. Wu leg., Wu 3704 (holotype, BJFC063820).

#### Etymology.

*Guangxiensis* (Lat.): referring to Guangxi Autonomous Region, China, where the species was collected.

#### Macrostructures.

Basidiomata annual, gregarious, gelatinous when fresh, hard when dry. Pilei 2–8 × 1–3 mm, small, conchoid or semicircular; pileal surface greyish-white (1B1) when fresh, pale gray (1C1) upon drying, convex, gelatinous and translucent, glabrous on the surface; margin straight, crenulate; context thin, transparent. Hymenophore concolorous with pilei, poroid, about 15–40 pores per basidiocarp; mature pores 0.2–0.5 mm diam., round to polygonal, larger near the base and smaller near the edge, the marginal pores often incomplete; tubes up to 0.3 mm long. Stipe absent.

#### Microstructures.

Basidiospores (6–)6.2–11 × (4–)4.1–9 μm, L = 8.75 μm, W = 6.17 μm, Q = 1.38–1.47 (n = 90/3), broadly ellipsoid, thin-walled, smooth, with guttules, faintly IKI+, CB–. Basidia 16–23 × 6–11 μm, clavate, inflated at apex, tapered towards base, with guttules, 4-spored, sterigmata 4–7 μm long; basidioles similar in shape to basidia, slightly smaller. Gloeocystidia and acanthocysts absent. Cheilocystidia occur along dissepiment edges, 21–35 × 13–17 μm, broom-shaped or irregular with small diverticulate projections on the sides and at the apex, thin-walled. Pileipellis hyphae interwoven, smooth, thin-walled, 2–6 μm in diam.; terminal cells irregular, with obtuse diverticulate projections on the sides and at the apex, orientated perpendicular to pileal surface. Tramal hyphae interwoven, smooth, loosely embedded in gelatinous matrix, 2–5 μm diam. Clamp connections present.

#### Additional specimen examined (paratype).

China • Guangxi Autonomous Region, Fangchenggang, Shangsi County, Shiwandashan National Forest Park, 21.9045°N, 107.9039°E, 281 m a.s.l.; 22 July 2025, on dead bamboo, F. Wu leg., Wu 3637 (BJFC063753); • Qinzhou, Bazhaigou Scenic Spot, 22.1049°N, 108.2543°E, 74 m a.s.l.; 23 July 2025, on dead bamboo, F. Wu leg., Wu 3705 (BJFC063821).

### Favolaschia microcarpa

Taxon classificationFungiAgaricalesMycenaceae

Yu H. Wang, Z.W. Zheng & F. Wu

B163B09D-E53E-5E69-BC12-ED5E5D868EDE

865073

Figs 5, 6, 7

#### Diagnosis.

It is distinguished by its very small (1–3 × 1–2 mm) and white basidiomata, broadly ellipsoid to subglobose basidiospores measuring 6.3–11 × 5.1–9 μm and inhabiting *Miscanthus* sp. and *Trachycarpus fortunei*.

**Figure 5.**
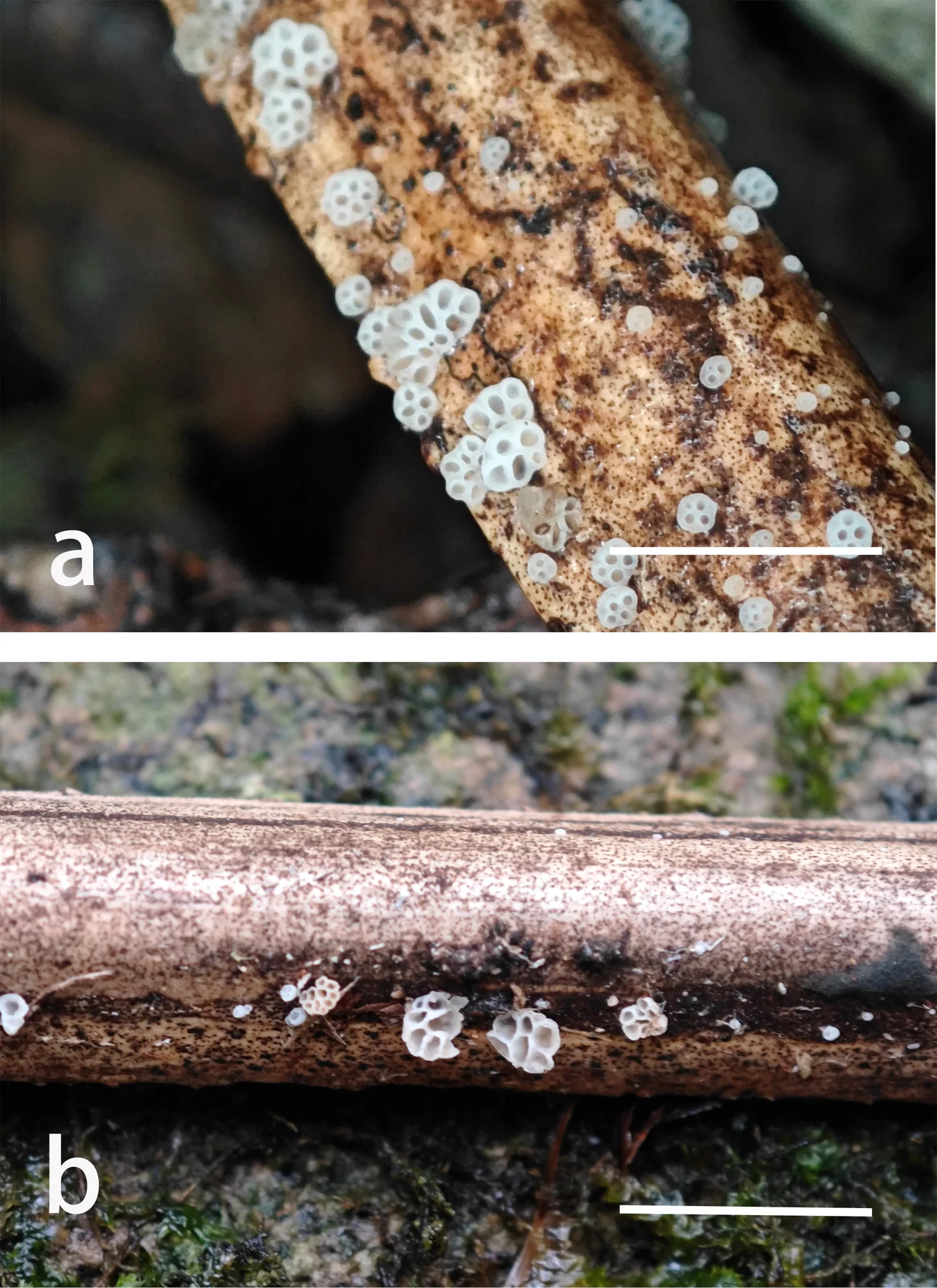
Basidiomata of *Favolaschia microcarpa*. **a**. Wu 3254 (holotype); **b**. Wu 4193. Scale bar: 1 cm.

**Figure 6.**
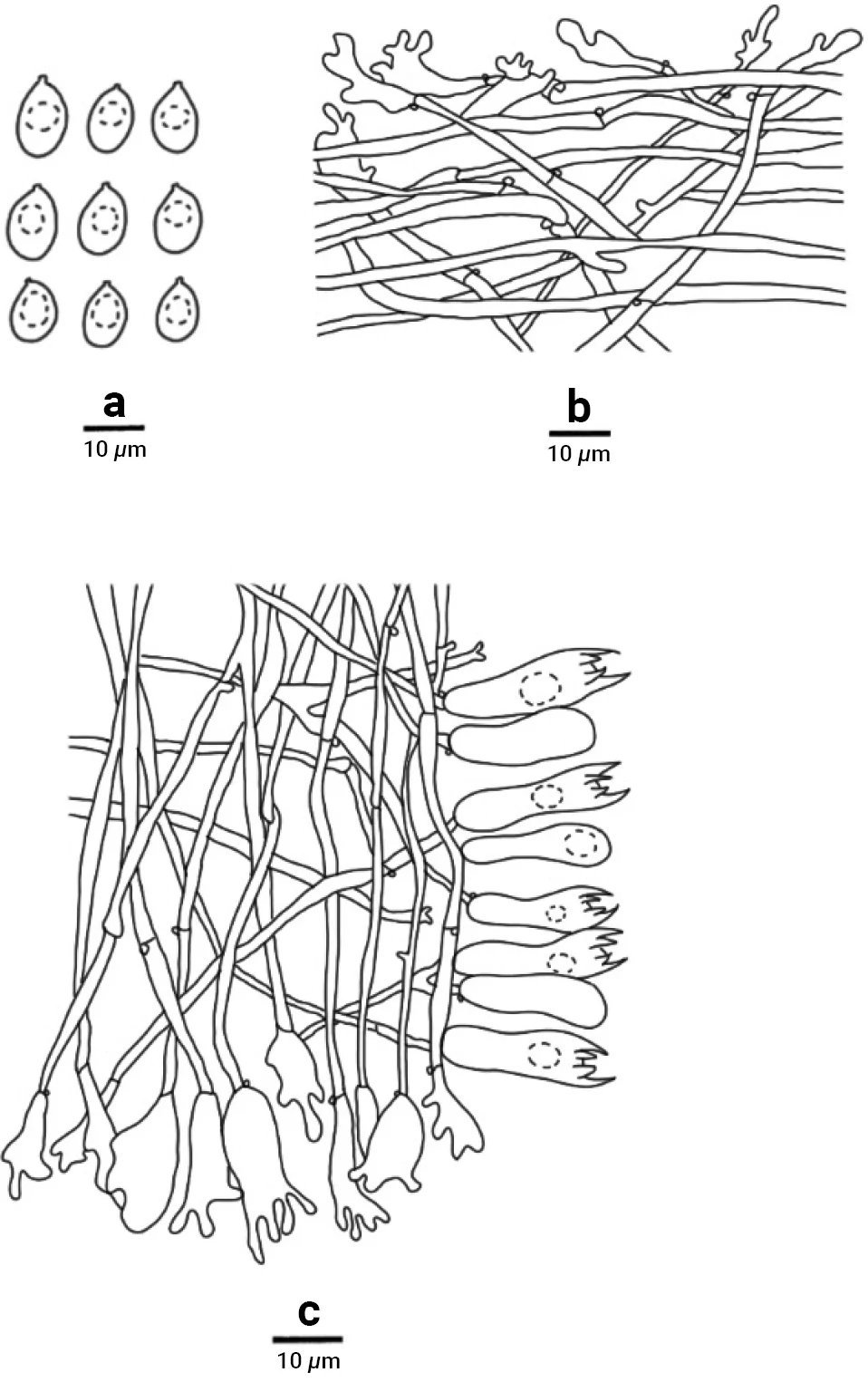
Microscopic structures of *Favolaschia microcarpa* (holotype). **a**. Basidiospores; **b**. Hyphae and terminal cells from pileipellis; **c**. A section of tube trama, including basidia, basidioles and cheilocystidia.

**Figure 7.**
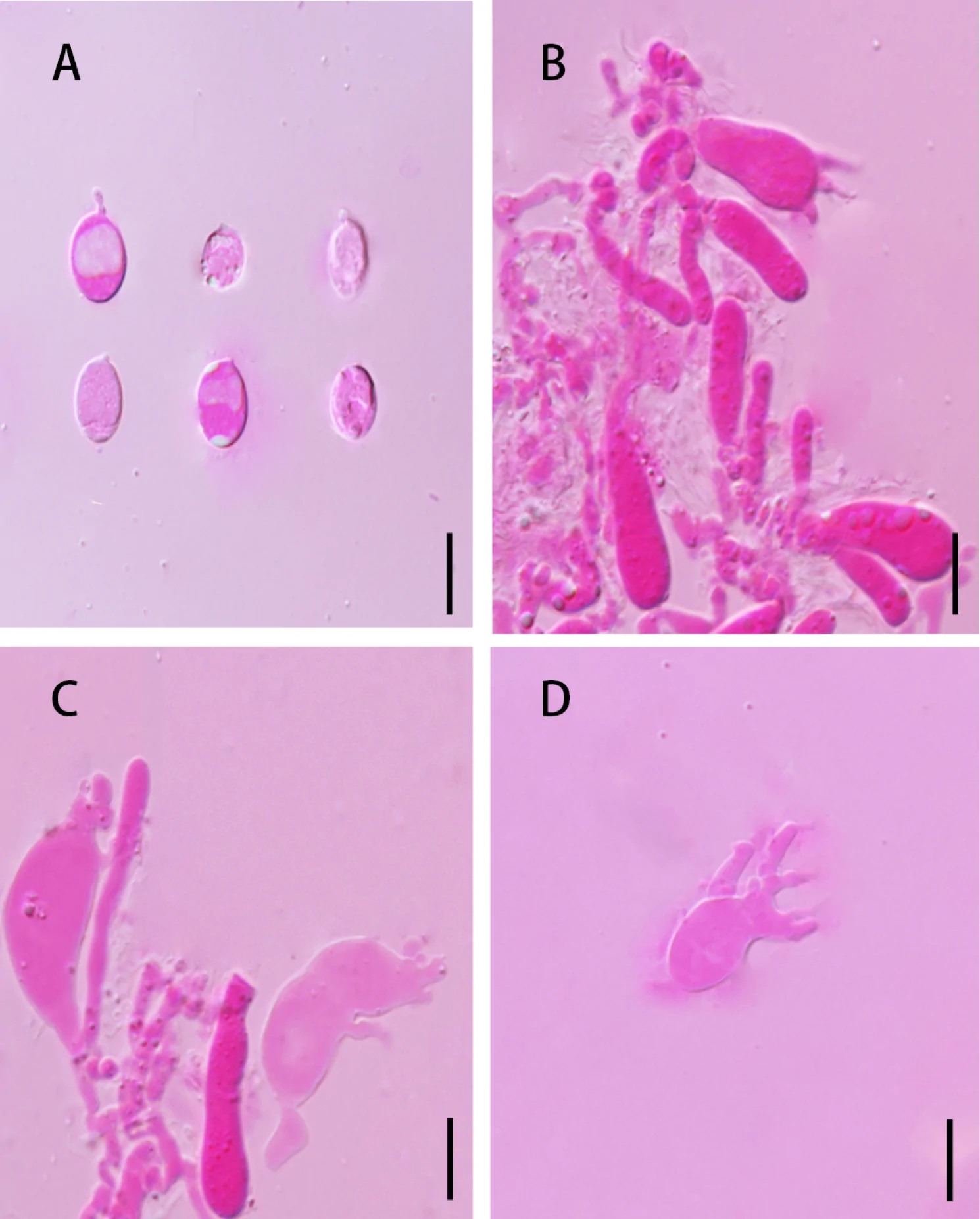
Microscopic structures of *Favolaschia guangxiensis* (holotype). **A**. Basidiospores; **B**. A section of tube trama, including basidia, basidioles; **C**. Cheilocystidia; **D**. Terminal cell of pileipellis.

#### Holotype.

China • Zhejiang Province, Hangzhou, Lin’an District, Dongtianmu Mountain Scenic Area, 30.3405°N, 119.5177°E; 460 m a.s.l.; 24 June 2025, on *Miscanthus*, F. Wu leg., Wu 3254 (holotype, BJFC063370).

#### Etymology.

*Microcarpa* (Lat.): referring to the very small basidiomata of the species.

#### Macrostructures.

Basidiomata annual, gregarious, gelatinous when fresh, hard when dry. Pilei 1–3 × 1–2 mm, conchoid or subcircular; pileal surface white (A1) when fresh, slightly shrunken and darkened upon drying, transparent with a reticulate pattern matching the pores below, glabrous; margin straight, crenulate, context thin, transparent. Hymenophore small, gelatinous, poroid, with 4–16 pores per basidiocarp; mature pores 0.2–0.5 mm in diam., round to polygonal, larger near the base and smaller near the edge, the marginal pores often incomplete; tubes up to 0.1 mm long. Stipe absent.

#### Microstructures.

Basidiospores (6–)6.3–11 × (5–)5.1–9 μm, L = 8.82 μm, W = 7.12 μm, Q = 1.18–1.30 (n = 90/3), broadly ellipsoid to subglobose, hyaline, thin-walled, smooth, with guttules, faintly IKI+, CB–. Basidia 12–23 × 5–9 μm, clavate, with guttules, 4-spored, sterigmata 5–9 μm long; basidioles similar in shape to basidia, slightly smaller. Gloeocystidia and acanthocysts absent. Cheilocystidia occur along dissepiment edges, 17–25 × 11–17 μm, broom-shaped or irregular with small diverticulate projections on the sides and at the apex, thin-walled. Pileipellis hyphae interwoven, smooth to diverticulate, thin-walled, 2–5 μm in diam.; terminal cells irregular with small diverticulate projections on the sides and at the apex, perpendicularly arranged to pileal surface. Tramal hyphae interwoven, smooth, loosely embedded in gelatinous matrix, 2–4 μm diam. Clamp connections present.

#### Additional specimen examined (paratype).

China • Zhejiang Province, Hangzhou, Linan District, East Tianmu Mountain Scenic Area, 30.3405°N, 119.5177°E; 461 m a.s.l.; 24 June 2025, on *Miscanthus*, F. Wu leg., Wu 3255 (BJFC063371). • West Tianmu Mountain National Nature Reserve, 30.3287°N, 119.4392°E; 487 m a.s.l.; 14 September 2025, on *Trachycarpus fortunei*, F. Wu leg., Wu 4193 (BJFC064309).

## Discussion

*Favolaschia* species were first recorded in China in the 20^th^ century (Liu 1994; Liu and Yang 1994). In recent years, the species diversity of *Favolaschia* in China has been continually refined by combining molecular phylogenetic methods (Liu 2020; Tolgor et al. 2021; Zhang and Dai 2021; Zhang et al. 2023, 2024; Lu et al. 2025). Two new species, *F. guangxiensis* and *F. microcarpa*, from southern China are described in the present study, which provides new data for a better understanding of the diversity of this genus.

*Favolaschia guangxiensis* is morphologically similar to *F. aulaxina* (Mont.) Singer and *F. flabelliformis* Q.Y. Zhang & Y.C. Dai by having gelatinous basidiomata and similar pilei in color. However, *F. aulaxina* differs from *F. guangxiensis* by its pores forming radially distant lamellae at edges and with a short stipe (Singer 1974), and *F. flabelliformis* differs from *F. guangxiensis* by its smaller pilei (2–3 × 1–2 mm vs. 2–8 × 1–3 mm), smaller pores (0.2–0.25 mm vs. 0.2–0.5 mm in diam.) and longer basidia (28.5–35 μm vs. 16–23 μm in length, Zhang et al. 2023). Phylogenetically, *F. guangxiensis* formed an independent and highly supported lineage closely related to *F. miscanthi* (Fig. 1). The two species share similar-sized pilei, but *F. miscanthi* has white pileal surface when fresh, oblong-ellipsoid basidiospores, longer basidia (32–45 μm vs. 16–23 μm), and narrower cheilocystidia (7–10 μm vs. 13–17 μm in diam.; Zhang et al. 2024). In addition, the two taxa show approximately 2% ITS sequence divergence, based on comparison with GenBank accession OR334879 (voucher specimen: Dai 24653).

Morphologically, *Favolaschia microcarpa* is similar to *F. rigida* Q.Y. Zhang & Y.C. Dai and *F. semicircularis* Q.Y. Zhang & Y.C. Dai. They all belong to F. sect. Anechinus subsect. Rubrinae and share pure white to white basidiomata when fresh and occur in southern China (Zhang et al. 2023). However, *Favolaschia rigida* differs from *F. microcarpa* by its obvious stipe, pileal surface with faintly pruinose when dry and the absence of cheilocystidia (Zhang et al. 2023). *Favolaschia semicircularis* is readily distinguished from *F. microcarpa* by its obvious stipe and longer basidia (24–32 μm vs. 12–23 μm in length, Zhang et al. 2023). In addition, *F. miscanthi*, which also occurs on *Miscanthus*, differs by its larger pilei (2–5 × 1.5–4 mm vs. 1–3 × 1–2 mm), oblong-ellipsoid basidiospores, and larger basidia (32–45 × 7–10 μm vs. 12–23 × 5–9 μm, Zhang et al. 2024). Phylogenetic analyses revealed that *F. microcarpa* formed an independent lineage closely related to *F. bannaensis* (Fig. 1). However, *F. bannaensis* can be distinguished by its larger pilei (2–5 × 1.5–4 mm vs. 1–3 × 1–2 mm), bigger basidia (30–38 × 7–10 μm vs. 12–23 × 5–9 μm), and inhabiting bamboo or rotten angiosperms (Zhang et al. 2023). Furthermore, the ITS sequence similarity between the *F. microcarpa* and the *F. bannaensis* (ON870498, voucher specimen: Dai 22589) is approximately 97%.

In recent years, with the continuous advancement of fungal taxonomic research, numerous new *Favolaschia* species, especially Favolaschia sect. Anechinus, have been gradually discovered in China, further enriching the species diversity of wood-inhabiting fungi in the country. The discovery of the new species enriches the known diversity of *Favolaschia* in eastern and southern China. It demonstrates that *Favolaschia* species can occur on diverse poaceous and palm substrates, including bamboo, *Trachycarpus* and *Miscanthus*. Future investigations are expected to reveal more taxa of this genus in subtropical China.

## Supplementary Material

XML Treatment for Favolaschia guangxiensis

XML Treatment for Favolaschia microcarpa

## Additional information

### Artificial Intelligence (AI) use

The authors accept full responsibility for the content of the manuscript, including the disclosure of any use of AI.

No AI tools were used in the preparation of this manuscript.

### Author ORCIDs

Y.-H. Wang https://orcid.org/0009-0006-2074-4746

Z.-W. Zheng https://orcid.org/0009-0006-8442-408X

Q.-X. Guan https://orcid.org/0000-0002-7072-080X

W.-Y. Li https://orcid.org/0009-0006-7782-6670

S.-B. Liu https://orcid.org/0000-0002-2275-3922

G.-Y. Zeng https://orcid.org/0009-0009-8609-1612

F. Wu https://orcid.org/0000-0002-1455-6486

### Data availability

All of the data that support the findings of this study are available in the main text or Supplementary Information.

## Supplementary materials

Supplementary material 1 All sequences analyzed in this study Data type fas https://binary.pensoft.net/file/1827696This dataset is made available under the Open Database License (http://opendatacommons.org/licenses/odbl/1.0/). The Open Database License (ODbL) is a license agreement intended to allow users to freely share, modify, and use this Dataset while maintaining this same freedom for others, provided that the original source and author(s) are credited. Yu-Han Wang, Zi-Wei Zheng, Qian-Xin Guan, Wan-Ying Li, Shu-Bin Liu, Guang-Yu Zeng, Fang Wu

## References

[B1] Bodensteiner P, Binder M, Moncalvo JM, Agerer R, Hibbett DS (2004) Phylogenetic rela-tionships of cyphelloid homobasidiomycetes. Molecular Phylogenetics and Evolution 33: 501–515. 10.1016/j.ympev.2004.06.007 15336682

[B2] Capelari M, Karstedt F, de Oliveira JJS (2013) *Favolaschia* in remnants of the Atlantic Forest, Brazil. Mycoscience 55(1): 12–20. 10.1016/j.myc.2013.03.004

[B3] Clémençon H (1977) Anatomie der Hymenomyceten (Anatomy of the *Hymenomycetes*). In: Flück-Wirth T (Ed.) Universität Lausanne, Lausanne, Switzerland.

[B4] Dong JH, Li Q, Yuan Q, Luo YX, Zhang XC, Dai YF, Zhou Q, Liu XF, Deng YL, Zhou HM, Muhammad A, Zhao CL (2024) Species diversity, taxonomy, molecular systematics and divergence time of wood-inhabiting fungi in Yunnan-Guizhou Plateau, Asia. Mycosphere 15(1): 1110–1293. 10.5943/mycosphere/15/1/10

[B5] Dong JH, Chen ML, Chen M, Li Q, Zhu YJ, Zhang XC, Zhou CQ, Li W, Muhammad A, Zhou HM, Jabeen S, Zhao CL (2025) Notes, outline, taxonomy and phylogeny of wood-in habiting *Agaricales*. Mycosphere 16(1): 2599–2711. 10.5943/mycosphere/16/1/16

[B6] Gillen K, Laessoe T, Kirschner R, Piepenbring M (2012) *Favolaschia* species (*Agaricales*, *Basidiomycota*) from Ecuador and Panama. Nova Hedwigia 96(1–2): 117–165. 10.1127/0029-5035/2012/0070

[B7] Hall TA (1999) BioEdit: A user-friendly biological sequence alignment editor and analysis program for Windows 95/98/NT. Nucleic Acids Symposium Series 41: 95–98.

[B8] Johnston PR, Whitton SR, Buchanan PK, Park D, Moncalvo JM (2006) The basidio­mycete genus *Favolaschia* in New Zealand. New Zealand Journal of Botany 44(1): 65–87. 10.1080/0028825X.2006.9513007

[B9] Kalyaanamoorthy S, Minh BQ, Wong TKF, von Haeseler A, Jermiin LS (2017) ModelFinder: Fast model selection for accurate phylogenetic estimates. Nature Methods 14(6): 587–589. 10.1038/nmeth.4285 PMC5453245 28481363

[B10] Katoh K, Rozewicki J, Yamada KD (2017) MAFFT online service: Multiple sequence alignment, interactive sequence choice and visualization. Briefings in Bioinformatics 20(4): 1160–1166. 10.1093/bib/bbx108 PMC6781576 28968734

[B11] Kornerup A, Wanscher JH (1978) Methuen handbook of color. 3^rd^ edn. E. Methuen and Co., Ltd., London, 252 pp.

[B12] Liu LN (2020) Taxonomy and molecular phylogeny of *Mycenaceae* in China. PhD Thesis, Jilin Agricultural University, Jilin, China.

[B13] Liu PG (1994) A supplementary notes on the *Laschia*-complex from southern and southeastern Yunnan. Yunnan Zhi Wu Yan Jiu 16: 243–247.

[B14] Liu PG, Yang ZL (1994) Studies of classification and geographic distribution on *Laschia* complex from the southern and southeastern Yunnan, China. Yunnan Zhi Wu Yan Jiu 16: 47–52.

[B15] Lu W, Chen X, Kumla J, Suwannarach N, Elgorban AM, Dai DQ, Tibpromma S, Karunarathna SC (2025) A new species of *Favolaschia* (*Agaricales*, *Mycenaceae*) from Yunnan Province, China. Phytotaxa 694(2): 144–160. 10.11646/phytotaxa.694.2.3

[B16] Magnago AC, Trierveiler-Pereira L, Neves MA (2013) Contributions towards the know­ledge of *Favolaschia* (*Mycenaceae*, *Agaricomycetes*) from Brazil. Mycosphere 4(6): 1071–1078. 10.5943/mycosphere/4/6/5

[B17] Moncalvo JM, Vilgalys R, Redhead SA, Johnson JE, James TY, Aime MC, Hofstetter V, Verduin SJW, Larsson E, Baroni TJ, Thorn RG, Jacobsson S, Clémencon H, Miller OK (2002) One hundred and seventeen clades of euagarics. Molecular Phylogenetics and Evolution 23(3): 357–400. 10.1016/S1055-7903(02)00027-1 12099793

[B18] Patouillard N (1887) Etude sur le genre *Laschia* Fr. Journal de Botanique. Premiere Année 15: 225–231.

[B19] Patouillard NT, Lagerheim G (1892) Champignons de l’Équateur. Pugillus II. Bulletin de la Société Mycologique de France 8: 113–140.

[B20] Petersen JH (1996) The Danish mycological society’s colour-chart. Greve.: Foreningen til Svampekundskabens Fremme, 6 pp.

[B21] Rambaut A (2018) Molecular evolution, phylogenetics and epidemiology. FigTree ver. 1.4.4 software. [Accessed October 2022]

[B22] Ronquist F, Huelsenbeck JP (2003) MrBayes 3: Bayesian phylogenetic inference undermixed models. Bioinformatics (Oxford, England) 19(12): 1572–1574. 10.1093/bioinformatics/btg180 12912839

[B23] Sayers EW, Cavanaugh M, Clark K, Pruitt KD, Sherry ST, Yankie L, Karsch-Mizrachi I (2024) GenBank 2024 Update, Nucleic Acids Research 52(1): 134–137. 10.1093/nar/gkad903 PMC10767886 37889039

[B24] Singer R (1945) The *Laschia*-complex (*Basidiomycetes*). Lloydia 8: 170–230.

[B25] Singer R (1974) A monograph of *Favolaschia*. Beihefte zur Nova Hedwigia 50: 1–108.

[B26] Song CG, Xu TM, Xu YH, Wang D, Zeng L, Fan XP, Sun YF, Cui BK (2025) Systematic revision, molecular phylogeny and divergence times of *Thelephorales (Basidiomycota)*. Mycosphere 16(1): 296–422. 10.5943/mycosphere/16/1/5

[B27] Stamatakis A (2006) RAxML-VI-HPC: Maximum Likelihood-based phylogenetic analy ses with thousands of taxa and mixed models. Bioinformatics 22(21): 2688–2690. 10.1093/bioinformatics/btl446 16928733

[B28] Tolgor B, Na Q, Liu LN (2021) A monograph of *Mycenaceae (Agaricales)* in China. Science press, Beijing, 326 pp.

[B29] Vilgalys R, Hester M (1990) Rapid genetic identification and mapping of enzymatically amplified ribosomal DNA from several *Cryptococcus* species. Journal of Bacteriology 172(8): 4238–4246. 10.1128/jb.172.8.4238-4246.1990 PMC213247 2376561

[B30] Wang CG, Dai YC, Kout J, Gates GM, Liu HG, Yuan Y, Vlasák J (2024) Multi–gene phylo­geny and taxonomy of *Physisporinus* (*Polyporales*, *Basidiomycota*). Mycosphere 15(1): 1455–1521. 10.5943/mycosphere/15/1/12

[B31] White TJ, Bruns T, Lee S, Taylor J (1990) Amplification and direct sequencing of fungal ribosomal RNA genes for phylogenetics. PCR Protocols: A Guide to Methods and Applications. Academic Press, New York, 315–322. 10.1016/b978-0-12-372180-8.50042-1

[B32] Wu F, Li SJ, Dong CH, Dai YC, Papp V (2020) The genus *Pachyma* (Syn. *Wolfiporia*) reinstated and species clarification of the cultivated medicinal mushroom “Fuling” in China. Frontiers in Microbiology 11: 590788. 10.3389/fmicb.2020.590788 PMC7793888 33424793

[B33] Wu F, Zhou LW, Vlasák J, Dai YC (2022) Global diversity and systematics of *Hymenochaetaceae* with poroid hymenophore. Fungal Diversity 113(1): 1–192. 10.1007/s13225-021-00496-4

[B34] Yang Y, Xu Y, Wang L, Jiang QQ, Su JQ, Li R, Zhou HM, Zhao CL (2025) Multigene phylogeny of seven wood-inhabiting fungal orders in *Basidiomycota*, and proposal of a new genus and thirteen new species. Mycosphere 16(1): 245–295. 10.5943/mycosphere/16/1/4

[B35] Zhang QY, Dai YC (2021) Taxonomy and phylogeny of the *Favolaschia calocera* complex (*Mycenaceae*) with descriptions of four new species. Forests 12(10): 1397. 10.3390/f12101397

[B36] Zhang QY, Liu HG, Papp V, Zhou M, Dai YC, Yuan Y (2023) New insights into the classification and evolution of *Favolaschia* (*Agaricales*, *Basidiomycota*) and its potential distribution, with descriptions of eight new species. Mycosphere 14(1): 777–814. 10.5943/mycosphere/14/1/10

[B37] Zhang QY, Liu HG, Li WY, Zhang X, Dai YC, Wu F, Bian LS (2024) Three new species of *Favolaschia* (*Mycenaceae*, *Agaricales*) from South China. MycoKeys 104: 71–89. 10.3897/mycokeys.104.117310 PMC11040199 38665969

[B38] Zhao H, Cui YJ, Guan QX, Wang K, Zhuang L, Zeng GY, Wei YL, Wu F, Yuan HS (2025) Global fungal diversity and distribution patterns within the order *Hymenochaetales* (*Agaricomycetes*, *Basidiomycota*). Mycosphere 16: 3257–3280. 10.5943/mycosphere/16/1/24

